# Institutional Housekeeping

**Published:** 1912-02-10

**Authors:** 


### INSTITUTIONAL HOUSEKEEPING.

(Workers' Questions and Criticism of every kind will be welcomed.)

### The Art of Buying.

### SUGARS.

The importance of sugar is so great that it behoves buyers to pay special consideration to its selection. The principal classes of sugar for domestic use necessary to consider are cane sugar and sugars corresponding to cane, spoken of chemically as sucroses. These include, amongst other varieties, beet and maple sugar. The chief varieties of raw cane sugar are Barbadoes, moist and very dark, and Demerara or Trinidad, of a golden colour with well-defined crystals and a fine flavour.

To ordinary consumers it is almost impossible to distinguish between cane and beet sugars, and the competition between the two has of late years become very acute. Pure cane icing-sugar is, however, to be preferred, as beet sugar, when subjected to heat, is apt to deteriorate in colour and render the icing less perfectly white. Maple sugar does not differ chemically from cane and beet sugars, but its commercial value is far below theirs. It is chiefly used for its characteristic and agreeable flavour, derived from the presence of certain substances, on which in all probability depend also its slightly laxative qualities. Sugar is now rarely adulterated, the only common addition being that of colouring-matter applied to the whitish beet crystals to make them resemble the bright straw tint of Demerara sugar.

One method of detecting low-class beet from cane sugar is to put some of the former into an infusion of tea without milk, when usually it will darken the colour. This is due to the combination of the tannin of the tea with a little iron sometimes found as an impurity in the less highly refined samples of beet sugar, but not in the purer kinds. One test for dyed sugar given by an expert is to place a little of the sugar in a white cup and pour on to it sufficient pure colourless dilute hydrochloric acid to cover it, and gently shake together. If the sugar be dyed the liquid will colour according to the dye used, if not it remains practically unchanged. Another test for Demerara sugar is to take a handful of it and squeeze it together, when if genuine Demerara and not beet yellow crystals, the particles will adhere owing to the presence of the syrup from which it was prepared. In the beet crystals this cohesion is not present, as owing to the high degree of refining necessary before beet sugar can be put on the market the particles are free from syrup. Occasionally raw sugars of a low class become infested with sugar mites. To detect these a sample of the sugar should be dissolved in warm water in a glass, when the mites will either adhere to the sides of it or float on the surface. Examination under a microscope will then easily identify the presence of these objectionable little insects. Sugars must be stored in a cool, perfectly dry place, either in well-closed stone jars or tins. The latter are best enamel-lined, as the tin coating of the interior soon becomes rusty.

---

### Questions and Answers on Practical Points.

Wanted a simple recipe for home-made yeast for a small institute situated far from any town or village.

The following is an excellent recipe for home-made yeast: for the first time of preparing it a little compressed yeast is needed, but not afterwards. Put nine pints of cold water into a saucepan, add to it three handfuls of dried hops (procurable from chemist or corndealer), and six large washed but unpeeled potatoes cut up coarsely. Boil these all slowly until the potatoes are soft, keeping the mixture well stirred. Mash them finely to remove lumps, and stand the mixture aside until it feels just warm to the hand. Next add half a pound of brown sugar, half an ounce of compressed yeast—mixed until liquid with one teaspoonful of sugar. Put ten ounces of flour into a basin, mix it smoothly with the above ingredients and pour all into wide mouthed jars or bottles. Tie down and keep in a cool place. Use half a pint to seven pounds of flour, shaking the jar before straining off some to use. After the first time save a little of the last yeast made to add to the mixture instead of the compressed yeast.

The manager of a soup-kitchen would like suggestions for preparing a cheap nutritious soup, to cost about 4d. per gallon.

Take 10½ gallons of cold water, six pounds of shin of beef, three pounds of split peas, two pounds of fine oatmeal, three pounds of onions, two pounds of carrots, one pound of dripping, one ounce of mixed herbs. The above recipe should cost under 4d. per gallon. If made regularly the split peas and oatmeal could be bought by the hundredweight, thus reducing the cost somewhat. Put the cold water into the boiler, add the bones, first chopping them and cutting any meat on them into small squares, add a tablespoonful of salt and allow them to soak for one hour. Heat slowly to boiling-point, then add the dripping, cleaned and chopped vegetables, peas, and the herbs tied in a bag. Boil gently for four hours. An hour before the cooking is finished add the oatmeal mixed smoothly and thinly with some cold water. When the peas are boiled to a soft mash, remove the herbs, season the soup carefully and it is ready. If liked, two stale quartern loaves may be cut up into cubes and added to the soup when serving it out.

